# Preventive effects of galcanezumab in adult patients with episodic or chronic migraine are persistent: data from the phase 3, randomized, double-blind, placebo-controlled EVOLVE-1, EVOLVE-2, and REGAIN studies

**DOI:** 10.1186/s10194-018-0951-2

**Published:** 2018-12-29

**Authors:** Stefanie Förderreuther, Qi Zhang, Virginia L. Stauffer, Sheena K. Aurora, Miguel J. A. Láinez

**Affiliations:** 1 Department of Neurology, Ludwig Maximilian University, Munich, Bavaria Germany; 2 Sanofi, Bridgewater, NJ USA; 3 Eli Lilly and Company, Lilly Corporate Center, Indianapolis, IN 46285 USA; 4 Hospital Clínico Universitario, Universidad Católica de Valencia, Valencia, Spain

**Keywords:** Galcanezumab, Migraine, Preventive, Persistence, Maintenance

## Abstract

**Background:**

Maintenance of effect following treatment with galcanezumab compared to placebo in adult patients with episodic or chronic migraine was evaluated.

**Methods:**

In 2 similarly designed studies of patients with episodic migraine (6 months) and 1 study of patients with chronic migraine (3 months), patients randomized in a 1:1:2 ratio received a subcutaneous injection of galcanezumab 120 mg/month (after an initial loading dose of 240 mg) or 240 mg/month or placebo. Maintenance of effect during the double-blind phase was evaluated based on a comparison of the percentages of galcanezumab- and placebo-treated patients with maintenance of 30, 50, 75, and 100% response (defined as ≥30, ≥50, ≥75, and 100% reduction from baseline in monthly migraine headache days [MHD]) at an individual patient level. Logistic regression analyses were used for between treatment comparisons.

**Results:**

A total of 1773 adult patients with episodic migraine (*n* = 444 for galcanezumab 120 mg; *n* = 435 for galcanezumab 240 mg; *n* = 894 for placebo for 2 studies pooled) and 1113 patients with chronic migraine (*n* = 278 for galcanezumab 120 mg; *n* = 277 for galcanezumab 240 mg; *n* = 558 for placebo) were evaluated. In patients with episodic migraine, ≥50% response was maintained in 41.5 and 41.1% of galcanezumab-treated patients (120 mg and 240 mg, respectively) for ≥3 consecutive months (until patient’s endpoint) and 19.0 and 20.5%, respectively, for 6 consecutive months and was significantly greater than the 21.4 and 8.0% of placebo-treated patients at ≥3 and 6 months consecutively (*P* < 0.001). Approximately 6% of galcanezumab-treated patients maintained ≥75% response all 6 months versus 2% of placebo-treated patients. Few galcanezumab-treated patients maintained 100% response. In patients with chronic migraine, 29% of galcanezumab-treated patients maintained ≥30% response all 3 months compared to 16% of placebo patients while ≥50% response was maintained in 16.8 and 14.6% of galcanezumab-treated patients (120 mg and 240 mg) and was greater than placebo (6.3%; *p* < 0.001). Few patients maintained ≥75% response.

**Conclusions:**

Treatment with galcanezumab 120 mg or 240 mg demonstrated statistically significant and clinically meaningful persistence of effect in patients with episodic migraine (≥3 and 6 consecutive months) and in patients with chronic migraine (for 3 months).

**Study identification and trial registration:**

Study Identification: EVOLVE-1 (I5Q-MC-CGAG); EVOLVE-2 (I5Q-MC-CGAH); REGAIN (I5Q-MC-CGAI)

**Trial Registration:**

ClinicalTrials.gov; NCT02614183 (EVOLVE-1); NCT02614196 (EVOLVE-2); NCT02614261 (REGAIN)

**Electronic supplementary material:**

The online version of this article (10.1186/s10194-018-0951-2) contains supplementary material, which is available to authorized users.

## Background

Galcanezumab is a humanized monoclonal antibody, indicated for the prevention of migraine, that binds to calcitonin gene-related peptide (CGRP) and prevents its biological activity without blocking the CGRP receptor [1]. The efficacy of galcanezumab was examined in 3 randomized, double-blind, placebo-controlled, Phase 3 studies of galcanezumab (120 and 240 mg/month) in patients with episodic (EVOLVE-1 and EVOLVE-2 6-month studies) or chronic (REGAIN 3-month study) migraine [2–4]. The mean monthly percentages of galcanezumab-treated patients with episodic migraine or chronic migraine that achieved ≥50% reduction in MHD was greater than the percentages of placebo-treated patients (60% versus 36% to 39% and 27% versus 15%, respectively) [2–4]. For patients with episodic migraine, galcanezumab-treated patients experienced approximately 4 fewer MHD/month (versus 2 with placebo) and patients with chronic migraine had approximately 5 fewer MHD/month (versus 3 with placebo) with a similar effect in both galcanezumab dose groups [2–4].

Data on current treatments for migraine prevention support that patients on recently approved and older treatments for migraine prevention do achieve a ≥ 50% level of response [5–7]. However, the important question of whether a ≥ 50% reduction in monthly MHD is maintained over time has not been sufficiently addressed for both episodic and chronic migraine [5, 8–10]. Further, can the additional responses of ≥30, ≥75, and 100% reduction in monthly MHD, also recognized to be clinically meaningful, be maintained [11–13]? For patients on a preventive treatment, this is a particularly important aspect since patients seek a medication with a consistent efficacy profile over time. Clinically, tachyphylaxis has been reported frequently by patients and physicians.

The current study evaluated data from the placebo-controlled EVOLVE-1 and EVOLVE-2 episodic migraine trials and the REGAIN chronic migraine trial and compared galcanezumab treatment to placebo in the maintenance of ≥30% (chronic only), ≥50, ≥75, and 100% (episodic only) response in the reduction of MHD from baseline.

## Methods

### Study design

Detailed descriptions of the study design for the 2 episodic migraine (6-month) and 1 chronic migraine (3-month) double-blind studies, have been reported separately (ClinicalTrials.gov NCT02614183, NCT02614196, and NCT02614261) [2–4]. Briefly, adult patients were randomized 1:1:2 and received subcutaneous injections of galcanezumab 120 mg/month (after a 240 mg initial loading dose) or 240 mg/month or placebo. Episodic migraine was defined as having between 4 and 14 MHD and at least 2 migraine attacks per month [2, 3, 14]. Chronic migraine was defined as having headache ≥15 days per month for ≥3 months and having features of migraine headache ≥8 days per month [4, 14]. The continuation or start of any additional migraine preventive treatments was not permitted; the exception for patients with chronic migraine was the use of topiramate or propranolol provided they entered trial on a stable dose. The ≥50, ≥75, and ≥ 100% response rates during the 6-month (episodic) or 3-month (chronic) study periods were key secondary objectives (adjusted for multiple testing) and response rates at each month (episodic and chronic studies) were secondary outcomes (not adjusted for multiple testing). The study protocols were reviewed and approved by the appropriate institutional review board for each of the study sites. The studies were conducted according to Good Clinical Practice and the Declaration of Helsinki guidelines. Patients provided written informed consent before undergoing study procedures. The trials are registered with ClinicalTrials.gov (NCT02614183, NCT02614196, and NCT02614261).

### Statistical method

Data from the 2 episodic migraine trials combined and 1 chronic migraine trial were included in the analysis. In these trials, a 30, 50, 75, and 100% response rate at each month was defined as the percentage of patients meeting a defined threshold (≥30, ≥50, ≥75, and 100%) in the reduction of the number of monthly MHD during the double-blind treatment period. Only patients with both a baseline and ≥ 1 month of non-missing post-baseline MHD values were included in the analysis. The evaluation of maintenance of effect during the double-blind treatment period was a comparison of the percentages of galcanezumab- and placebo-treated patients with maintenance of ≥30, ≥50, ≥75, and 100% response at the individual patient level. In the episodic studies, maintenance of response was calculated for those with at least 3 months (until patient’s endpoint) and 6 consecutive months and 3 consecutive months for chronic migraine. A logistic regression analysis was used for between-treatment group comparisons. At each month, a cumulative 50% maintenance of response was also calculated and defined as patients with ≥50% response at a specific month (or before) and all subsequent months. For repeated binary data of monthly ≥50% response and cumulative ≥50% sustained response, a categorical, pseudolikelihood-based repeated measures model was implemented with SAS PROC GLIMMIX [15]. Two-sided *p*-values were calculated and compared with significance level of 0.05.

## Results

### Patient disposition

Data from the episodic migraine trials were from 1773 adult patients with episodic migraine treated with 120 mg galcanezumab (*n* = 444), 240 mg galcanezumab (*n* = 435), or placebo (*n* = 894). Data from the chronic migraine trial were from 1113 patients with chronic migraine treated with 120 mg galcanezumab (*n* = 278), 240 mg galcanezumab (*n* = 277), or placebo (*n* = 558). Baseline demographics and disease characteristics of the episodic and chronic migraine populations show that over 80% were female, over 74% were white, had a mean age of 40 years, and had migraine disease duration of 20 years. As permitted by protocol for the chronic migraine trial, concomitant use of topiramate or propranolol during the double-blind phase, across all treatment groups, occurred in 10.3 and 3.6% of patients, respectively. At baseline, the mean MHD/month was 9.1 for episodic migraine and 19.3 for chronic migraine. The mean baseline Migraine Disability Assessment score for patients treated with galcanezumab or placebo was 33.1 for episodic migraine and 65.8 (galcanezumab) and 68.7 (placebo) for chronic migraine and was reflective of severe (episodic) and very severe (chronic) migraine disability. The mean baseline Migraine-Specific Quality of Life Questionnaire Role Function-Restrictive subdomain score for patients treated with galcanezumab or placebo with episodic migraine was 51.1 and 52.1, respectively, and with chronic migraine was 39.1 and 38.4, respectively. Patients with chronic migraine had greater functional impairment than patients with episodic migraine (Table 1).

**Table 1:** Patient demographics and disease characteristics of galcanezumab -treated patients from episodic and chronic migraine trials

Variables	Episodic Migraine Studies^a^		Chronic Migraine Study^b^	
	Galanezumab^a^ *N* = 879	Placebo *N* = 894	Galcanezumab^b^ *N* = 555	Placebo *N* = 558
Age, years, mean (SD)	40.7 (11.4)	41.9 (11.4)	40.4 (12.2)	41.6 (12.1)
Female, n (%)	744 (84.6)	755 (84.5)	463 (83.4)	483 (86.6)
Race white, n (%)	652 (74.2)	681 (76.2)	447 (80.7)	432 (77.4)
Ethnicity not Hispanic or Latino^c^, n (%)	664 (78.4)	677 (79.4)	387 (74.0)	401 (76.7)
Geographic region, n (%)				
North America	647 (73.6)	657 (73.5)	320 (57.7)	321 (57.5)
Europe	119 (13.5)	122 (13.7)	138 (24.9)	140 (25.1)
Other	113 (12.9)	115 (12.9)	97 (17.5)	97 (17.4)
Body mass index, kg/m^2^ mean (SD)	27.6 (5.5)	27.6 (5.5)	26.5 (5.4)	26.9 (5.6)
Migraine disease duration, years, mean (SD)	20.1 (12.2)	20.5 (12.5)	20.2 (12.7)	21.9 (12.9)
Migraine headache days/month, mean (SD)	9.1 (2.9)	9.1 (3.0)	19.3 (4.4)	19.6 (4.6)
MHD/month with acute medication use, mean (SD)	7.4 (3.4)	7.5 (3.4)	14.8 (6.3)	15.5 (6.6)
Headache days/month, mean (SD)	10.7 (3.7)	10.6 (3.4)	21.3 (4.0)	21.5 (4.1)
Migraine with aura, n (%)	467 (53.1)	471 (52.7)	294 (53.0)	310 (55.6)
Prior preventive treatment in past 5 years, n (%)	559 (63.6)	555 (62.1)	431 (77.7)	435 (78.0)
Failed ≥ 2 preventives in past 5 years, n (%)	88 (10.0)	85 (9.5)	165 (29.7)	163 (29.2)
MIDAS total, mean (SD)	33.1 (28.2)	33.1 (29.3)	65.8 (57.3)	68.7 (57.4)
MSQ RF-R, mean (SD)	51.1 (16.1)	52.1 (15.6)	39.1 (17.3)	38.4 (17.2)
PGI-S, mean (SD)	4.3 (1.2)	4.3 (1.2)	4.8 (1.3)	4.9 (1.2)

*Abbreviations*: *MHD* migraine headache days, *MIDAS* Migraine Disability Assessment, *MSQ* Migraine-Specific Quality of Life Questionnaire version 2.1, *PGI-S* Patient Global Impression of Severity, *RF-R* Role Function-Restrictive, *SD* standard deviation

^a^Pooled data from two parallel 6-month trials in patients with episodic migraine

^b^3-month trial

^c^Not all patients reported ethnicity data

### Proportions of patients with ≥50% response

The model-estimated proportions of patients with episodic migraine achieving ≥50% response were significantly greater for both galcanezumab dose groups compared to placebo starting at Month 1 (*p* < 0.001) and at each month after (*p* < 0.001), as well as overall across 6 months (*p* < 0.001) (Table 2). For patients with chronic migraine, the model-estimated proportions of patients achieving ≥50% response was significantly greater for both dose groups compared to placebo starting at Month 1 (*p* < 0.001) and at each month after (*p* ≤ 0.004), as well as overall across 3 months (*p* < 0.001) (Table 2). The absolute values for the proportions of patients with episodic or chronic migraine that achieved ≥50% response were very similar to the estimated values proportions and are shown in Additional file 1: Table S1.

**Table 2:** Model-estimated proportion of patients with episodic and chronic migraine with ≥ 50% response

Response rate	Galcanezumab 120 mg (*N* = 436)	Galcanezumab 240 mg (*N* = 428)	Placebo (*N* = 875)
Episodic migraine			
Overall 6 months	60.8%	58.7%	37.2%
Odds ratio (95% CI)	2.6 (2.2, 3.1)^*^	2.4 (2.0, 2.8)^*^	
Month 1, %	50.9%	47.2%	23.8%
Odds ratio (95% CI)	3.3 (2.6, 4.2)^*^	2.9 (2.2, 3.7)^*^	
Month 6, %	65.7%	63.2%	44.3%
Odds ratio (95% CI)	2.4 (1.9, 3.1)^*^	2.2 (1.7, 2.8)^*^	
Chronic migraine	Galcanezumab 120 mg (*N* = 273)	Galcanezumab 240 mg (*N* = 274)	Placebo (*N* = 538)
Overall 3 months	27.6%	27.5%	15.4%
Odds ratio (95% CI)	2.1 (1.6, 2.8)^*^	2.1 (1.6, 2.8)^*^	
Month 1, %	23.7%	21.2%	9.9%
Odds ratio (95% CI)	2.8 (1.9, 4.2)^*^	2.5 (1.7, 3.6)^*^	
Month 3, %	31.9%	34.0%	22.4%
Odds ratio (95% CI)	1.6 (1.2, 2.3)^†^	1.8 (1.3, 2.5)^*^	

*Abbreviations*: *CI* confidence interval

^*^p < 0.001 versus placebo

^†^*p* ≤ 0.004 versus placebo

### Maintenance of response

**Fig. 1:**
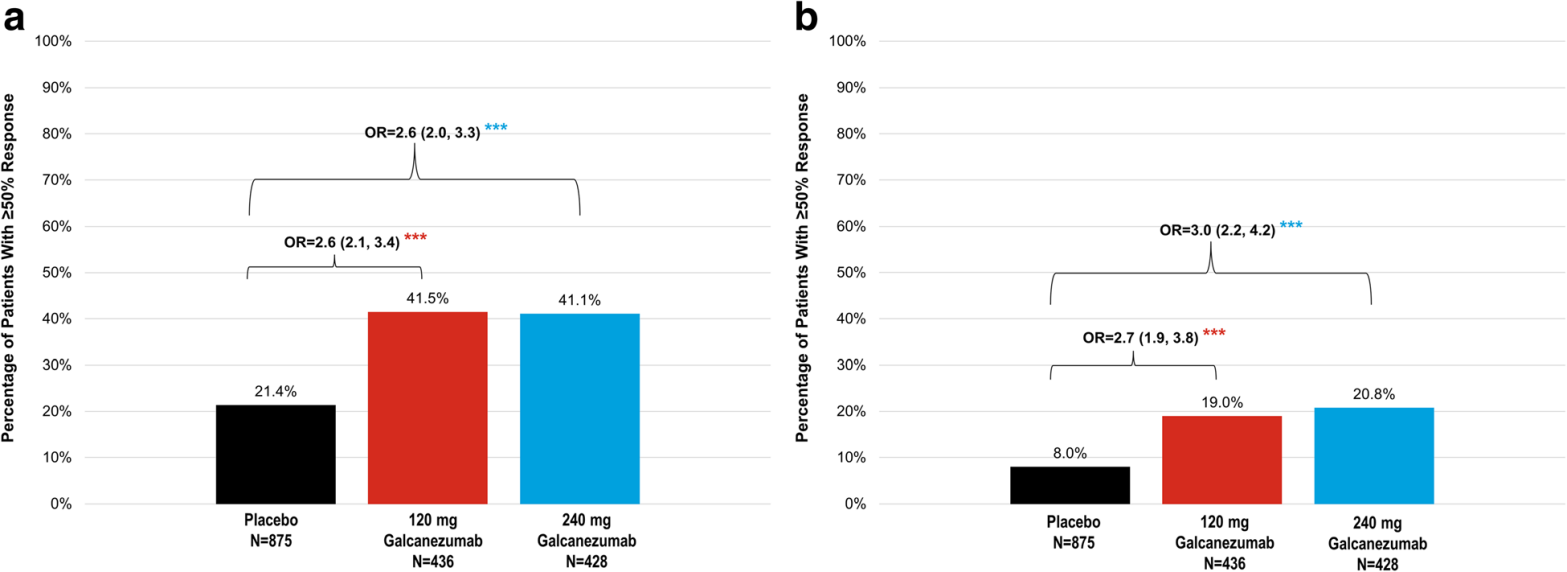
Episodic migraine: proportions of patients with maintenance of ≥ 50% response for ≥ 3 consecutive months until patient’s endpoint and response from month 1 to month 6. **a** maintenance of ≥ 50% response for ≥ 3 consecutive months. **b** maintenance of ≥ 50% response from month 1 to month 6. Abbreviations: OR = odds ratio. *** = *p* < 0.001

Significantly more patients with episodic migraine treated with galcanezumab in both dose groups (approximately 41%) maintained response of ≥50% fewer MHD for ≥3 consecutive months until patient’s endpoint compared to placebo (21%). Over Month 1 to Month 6, approximately 20% of galcanezumab-treated patients in both dose groups maintained a response of ≥50% fewer MHD that was significantly greater than placebo (8%) (Fig. 1).

**Fig. 2:**
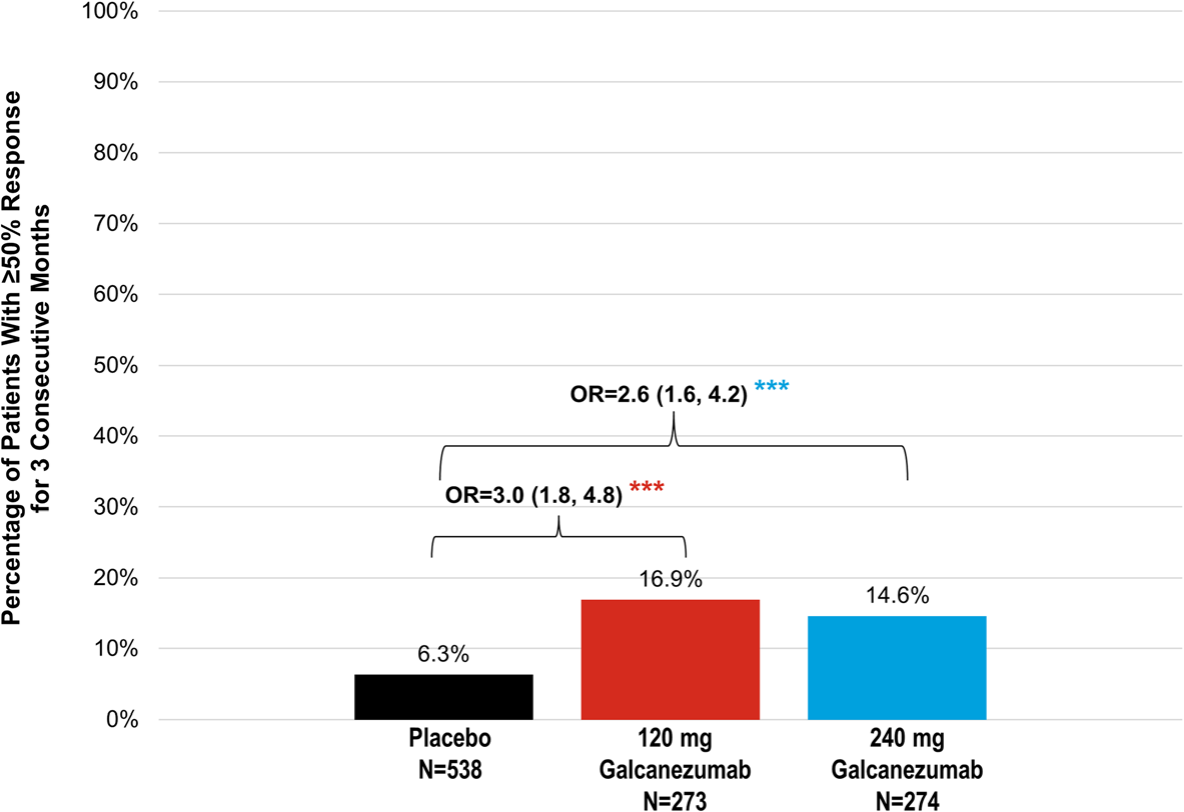
Chronic migraine: proportions of patients with maintenance of ≥ 50% response for 3 consecutive months. Abbreviations: OR = odds ratio. *** = *p* < 0.001

In patients with chronic migraine, significantly more patients in both the 120 mg (17%) and 240 mg (15%) galcanezumab dose groups maintained a response of ≥50% fewer MHD for 3 consecutive months compared to placebo (6%) (Fig. 2). The difference between dose groups for either episodic or chronic migraine in the proportions of patients with maintenance of response was not significant.

The model-estimated proportions of patients with episodic migraine who maintained a response of ≥75 and 100% and those patients with chronic migraine who maintained a response of ≥30 and ≥ 75% are shown in (Table 3). The proportions of galcanezumab-treated patients with episodic migraine who maintained a response of ≥75% fewer MHD for 3 consecutive months until patient’s endpoint (19.7 and 21.3%) was significantly greater than placebo (8.5%; *p* < 0.001). While fewer, 6% of galcanezumab-treated patients maintained a response of ≥75% fewer MHD for all 6 months compared with 1.8% of placebo-treated patients. A small percentage of patients maintained 100% response for 3 consecutive months and very few maintained 100% response for all 6 months. The proportions of galcanezumab-treated patients with chronic migraine who maintained a response of ≥30% fewer MHD for all 3 months (29%) was significantly greater than placebo (16.4%; *p* < 0.001). The proportions of patients who maintained ≥75% fewer MHD for all 3 months was not different between the galcanezumab and placebo groups.

**Table 3:** Model-estimated proportion of patients with episodic and chronic migraine with maintained response

Response rate	Galcanezumab 120 mg (*N* = 436)	Galcanezumab 240 mg (*N* = 428)	Placebo (*N* = 875)
Episodic migraine^a^			
75% Response ≥3 consecutive months^b^	19.7%	21.3%	8.5%
Odds ratio (95% CI)	2.7 (1.9, 3.7)^*^	2.9 (2.1, 4.1)^*^	
75% Response all 6 months	6.2%	6.8%	1.8%
Odds ratio (95% CI)	3.5 (1.9, 6.3)^*^	3.9 (2.1, 6.9)^*^	
100% Response ≥3 consecutive months^b^	3.7%	6.5%	2.7%
Odds ratio (95% CI)	1.4 (0.7, 2.5)	2.5 (1.4, 4.2)^*^	
100% Response all 6 months	0.7%	1.4%	0.2%
Odds ratio (95% CI)	2.9 (0.8, 10.4)	5.3 (1.6, 17.1)^†^	
Chronic migraine^c^	Galcanezumab 120 mg (*N* = 273)	Galcanezumab 240 mg (*N* = 274)	Placebo (*N* = 538)
30% Response all 3 months	29.3%	29.2%	16.4%
Odds ratio (95% CI)	2.1 (1.5, 3.0)^*^	2.1 (1.5, 3.1)^*^	
75% Response all 3 months	2.6%	2.9%	2.0%
Odds ratio (95% CI)	1.3 (0.6, 3.1)	1.5 (0.6, 3.4)	

*Abbreviations*: *CI* confidence interval

^a^6-month study

^b^Maintained response until patient’s endpoint of the 6-month, double-blind period

^c^3-month study

^*^p < 0.001 versus placebo

^†^ *p* = 0.006 versus placebo

### Characterization of patients with episodic migraine with ≥50% response at month 1

Among the 50.9 and 47.2% of galcanezumab 120 mg- and 240 mg-treated patients with episodic migraine who met ≥50% response at Month 1, the average reduction in MHD over the remaining 5 months of the double-blind phase was 66.6 and 71.5%. Of the 23.8% of placebo-treated patients with ≥50% response at Month 1, the average reduction in MHD was 63.2%. Further, among only those with ≥50% response at Month 1, 85.4 and 92.4% of patients in the 2 galcanezumab dose groups and 80.7% in the placebo group averaged at least a 40% response over the remaining 5 months. Moreover, 80.8 and 85.4% of patients in the 2 galcanezumab groups respectively and 71.1% of patients in the placebo group averaged at least ≥50% response over the remaining 5 months.

### Cumulative and onset of maintenance of response

**Fig. 3:**
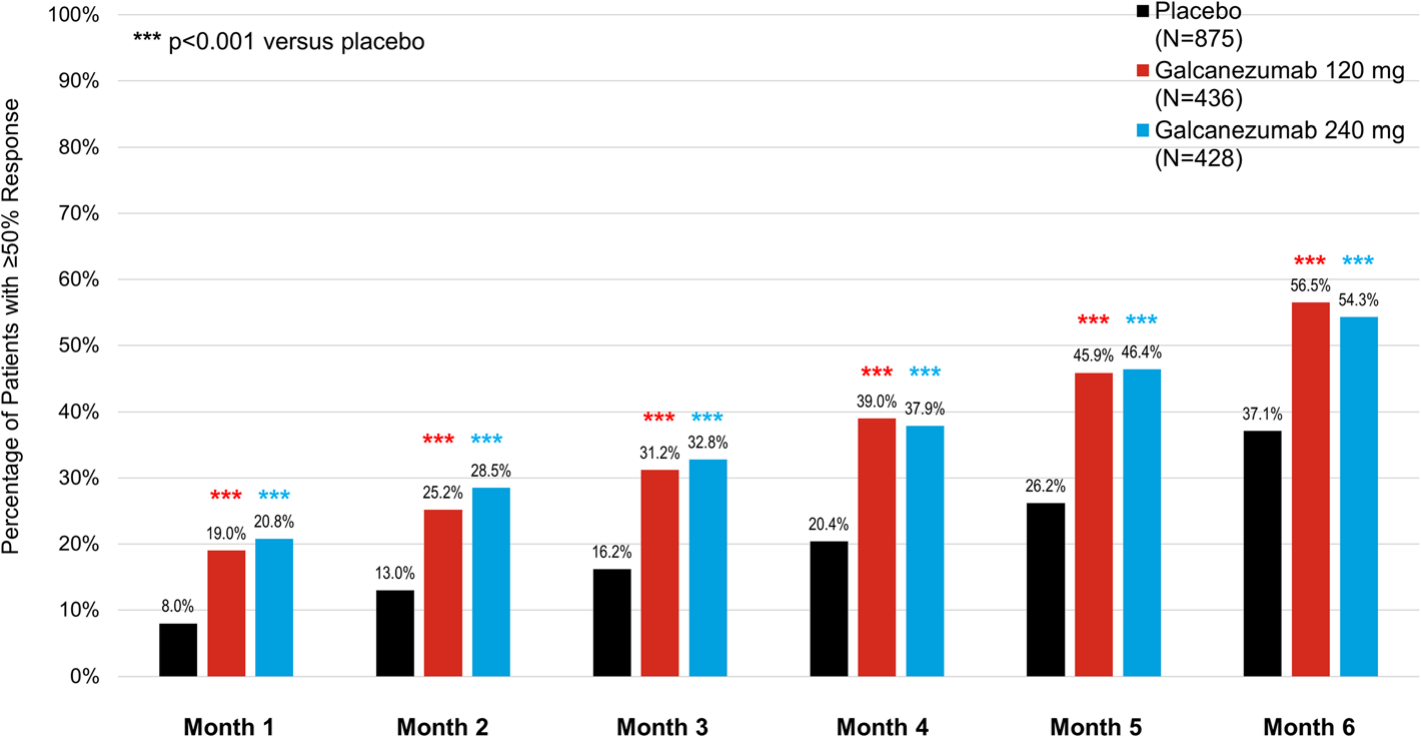
Cumulative maintained ≥ 50% response: percentages of patients with episodic migraine who reached ≥ 50% response at or before each month and all subsequent months

The cumulative maintenance of response is defined as individual patients who met ≥50% response starting at a given month (or before) and then all the months subsequent. The proportions of patients with episodic migraine in the galcanezumab 120 mg and 240 mg groups achieving cumulative maintenance of ≥50% response was superior to placebo at every month of the 6-month double-blind phase (*p* < 0.001) (Fig. 3). For example, 31% of patients with episodic migraine treated with galcanezumab 120 mg reached ≥50% at or before Month 3 and maintained that response in the subsequent months (Month 4 to Month 6). The difference between the galcanezumab dose groups for cumulative maintenance of response was not significant.

The onset of ≥50% maintenance of response and the percentage of patients with episodic or chronic migraine who reached ≥50% response at the specific month (but not before) and then maintained that response in the subsequent months of treatment is shown in Table 4. To illustrate the onset for patients with episodic migraine, 19, 6, and 6% of patients treated with galcanezumab 120 mg reached ≥50% response at Months 1, 2, and 3, respectively, and maintained that response in the subsequent months of treatment (Months 2 to 6, Months 3 to 6, and Month 4 to 6, respectively). Across the 6-month double-blind phase, onset of maintenance of response occurred for approximately 4 to 21% of galcanezumab-treated patients with highest rate occurring in Month 1. In all but one time point, the proportions of patients were greater in the galcanezumab treatment groups than the placebo group at each month. The exception was at 6 months where the proportions of patients were similar between the galcanezumab 120 mg (10.6%) and placebo (10.9%) groups.

**Table 4:** Onset of 50% maintenance of response in patients with episodic and chronic migraine: percentage of patients reach 50% response at each month and all the subsequent months

	Episodic migraine			Chronic migraine		
	Galcanezumab 120 mg (*N* = 436)	Galcanezumab 240 mg (*N* = 428)	Placebo (*N* = 875)	Galcanezumab 120 mg (*N* = 273)	Galcanezumab 240 mg (*N* = 274)	Placebo (*N* = 538)
Month 1	19.0%	20.8%	8.0%	14.8%	12.7%	5.6%
Month 2	6.2%	7.7%	5.0%	4.5%	6.6%	6.2%
Month 3	6.0%	4.3%	3.2%	10.9%	13.1%	9.0%
Month 4	7.8%	5.1%	4.2%	N/A	N/A	N/A
Month 5	6.9%	8.5%	5.8%	N/A	N/A	N/A
Month 6	10.6%	7.9%	10.9%	N/A	N/A	N/A

*Abbreviations*: *N/A* not applicable

**Fig. 4:**
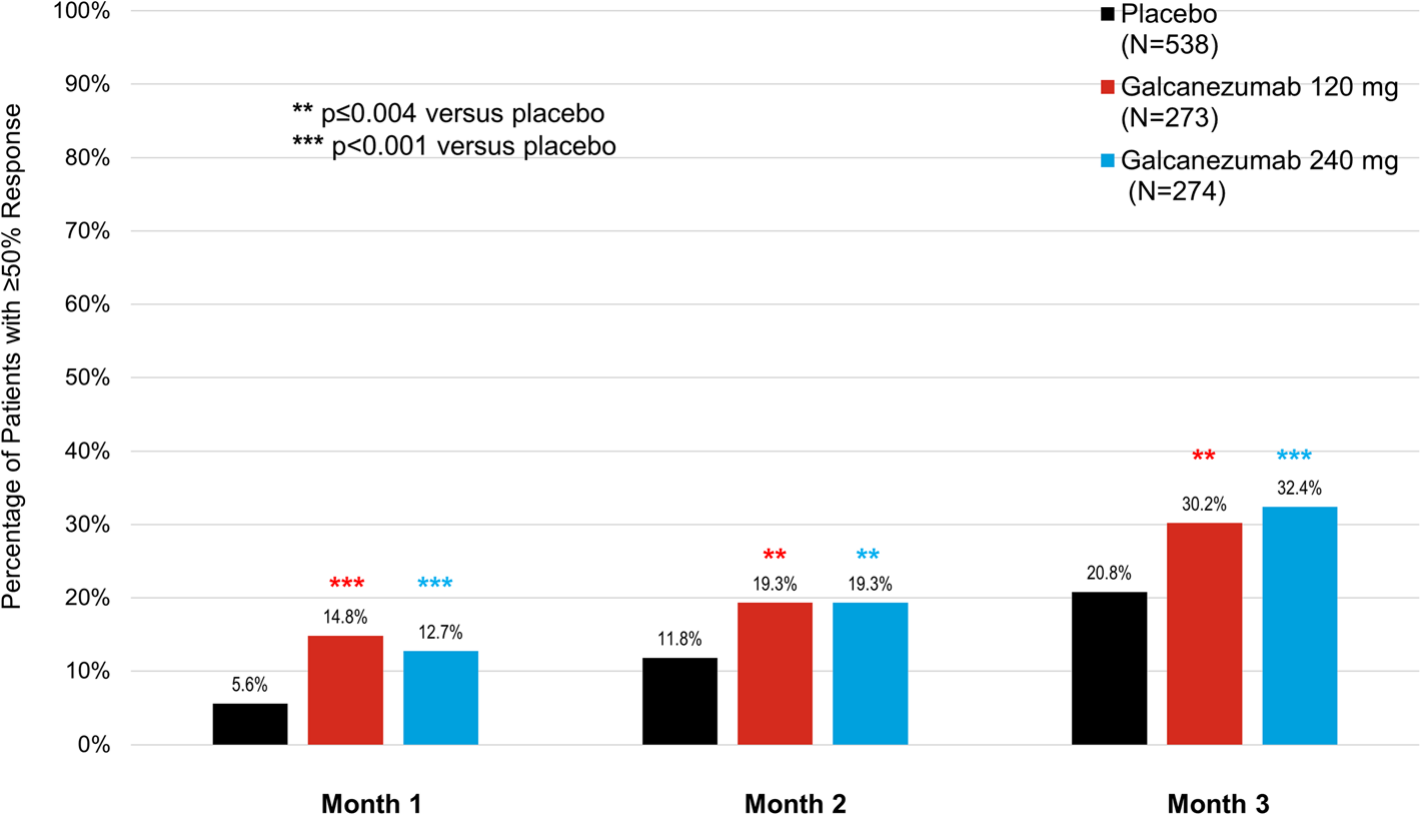
Cumulative maintained ≥ 50% response: percentages of patients with chronic migraine who reached ≥ 50% response at or before each month and all subsequent months

In patients with chronic migraine, the proportions of patients treated with galcanezumab 120 mg or 240 mg achieving cumulative maintenance of ≥50% response was superior to placebo at every month of the 3-month double-blind phase (*p* < 0.001). At Month 3 or before, approximately 30% of patients in the galcanezumab treatment groups achieved and maintained ≥50% response (Fig. 4). The difference between the galcanezumab dose groups for cumulative maintenance of response was not significant. To illustrate the onset of the maintenance of ≥50% response for patients with chronic migraine (Table 4), 15, 5, and 11% of patients treated with galcanezumab 120 mg reached ≥50% response at Month 1, 2, and 3, respectively.

### Safety and tolerability

The most commonly reported treatment-emergent adverse events (TEAE) were injection site-related pain, reaction, erythema, pruritus, and swelling. Discontinuation due to an injection site-related TEAE was low (< 0.5% across all 3 trials). There were no significant differences between galcanezumab and placebo in changes in vital signs and blood pressure. The safety profile between the 120 mg and 240 mg doses were similar [2–4].

## Discussion

Treatment with galcanezumab 120 mg or 240 mg demonstrated statistically significant and clinically meaningful maintenance of effect in patients with episodic migraine (≥3 consecutive months until patient’s endpoint and 6 consecutive months) or chronic migraine (3 months). Starting at Month 1, about 20% of galcanezumab-treated patients (either dose group) with episodic migraine had a sustained response of ≥50% reduction of MHD over 6 months; about 41% of patients maintained ≥50% response over ≥3 months. Among only the galcanezumab-treated patients who had a ≥ 50% reduction of MHD in Month 1, an average reduction of MHD of ≥40 and ≥ 50%, was achieved by 89 and 83% of patients, respectively, in the remaining 5 months of treatment suggesting minimal loss of efficacy among Month 1 responders. In galcanezumab-treated patients with chronic migraine, about 15% showed a ≥ 50% reduction of MHD over 3 consecutive months. Sustained efficacy was also observed in the placebo groups of patients with episodic and with chronic migraine; however, the placebo response was always significantly inferior to galcanezumab treatment. For example, galcanezumab-treated patients with episodic migraine were well over 2 times more likely than placebo-treated patients to achieve a sustained ≥50% response at 6 months and overall. Similarly, galcanezumab-treated patients with chronic migraine were twice as likely than placebo-treated patients to achieve a sustained ≥50% response at 3 months and overall. Several studies have shown the importance of expectation for the size of the placebo response and so a relatively high placebo response, typical for controlled treatment studies in migraine, was not an unexpected observation in our analysis [16–18]. The placebo response rate is likely a result of intensive patient care within the setting of a study. Regardless, the importance of this analysis is based on the fact that responders do not develop tachyphylaxis, for example, by up-regulation of other mediators of neurovascular inflammation.

Studies with monoclonal antibodies have shown sustained levels of 50% as well as 75 and 100% response in patients with episodic or chronic migraine [10]. Based on pre-specified analyses for our study, about 41% of galcanezuamb-treated patients with episodic migraine maintained ≥50% response for ≥3 consecutive months until patient’s endpoint and is a clinically relevant finding. In the additional post-hoc analysis of assessing the cumulative and onset of maintenance of ≥50% response, most patients reach ≥50% response and all subsequent months starting at Month 1, with approximately similar percentages of patients reaching ≥50% response and all subsequent months starting at Month 2, 3, 4, 5, and 6. These findings were generally consistent in the chronic migraine study although the percentages of patients with maintenance of response were generally lower given the higher baseline number of MHD. The 50% responder rate has been used as a secondary end-point in other trials, for example, with topiramate and botulinum toxin, in episodic and chronic migraine [5, 7, 19]. Comparison of those results to our data are difficult because only the 50% responder rate at the end of the double-blind period or at the end of cycle with botulinum toxin without any monthly and maintenance of response analysis was published.

Early and sustained response to preventive treatment is of special relevance. There is some evidence that improvement with preventive treatment at 3 months might be a predictor of persistent remission [20]. In addition, there is emerging evidence that more severely affected patients with a history of medication overuse, a high frequency of migraine attacks, and previously ineffective preventive treatments require long-term preventive treatment to maintain a reduced attack frequency, even after withdrawal of the preventive medication [21–23].

The maintenance of effect in patients with episodic or chronic migraine were similar between the galcanezumab 120 mg and 240 mg dose. This finding is consistent with previous reports in which there were no meaningful differences in efficacy measures between the galcanezumab doses and as such, the recommended dose is 120 mg after an initial loading dose of galcanezumab 240 mg (given as two 120 mg injections) [2–4].

One of the limitations of this paper is that the response rate was defined based on the primary efficacy measure, reduction in the number of MHD only. Additional analyses could be conducted in further publications to assess the response rate based on other efficacy measures, such as days of acute medication use, and measures of function and disability.

## Conclusions

Treatment with galcanezumab 120 mg or 240 mg demonstrated statistically significant and clinically meaningful maintenance of effect in patients with episodic migraine (at least 3 and 6 consecutive months) and in patients with chronic migraine (for 3 months).

## Additional file

Additional file 1: **Table S1.** Absolute proportion of patients with episodic and chronic migraine with ≥50% response. (DOCX 48 kb)

## Abbreviations

CGRP: Calcitonin gene-related peptide
MHD: Migraine headache days
