# Supplementary material for: Preventive effects of galcanezumab in adult patients with episodic or chronic migraine are persistent: data from the phase 3, randomized, double-blind, placebo-controlled EVOLVE-1, EVOLVE-2, and REGAIN studies
Source: J Headache Pain. 2018 Dec 29;19(1):121. doi: 10.1186/s10194-018-0951-2 (PMC6755564; doi:10.1186/s10194-018-0951-2)
Supplement: Supplementary file 1 — Table S1. Absolute proportion of patients with episodic and chronic migraine with ≥50% response. (DOCX 48 kb) [file 10194_2018_951_MOESM1_ESM.docx]

## Table S1. Absolute Proportion of Patients With Episodic and Chronic Migraine With ≥50% Response

| **Response rate** | **Galcanezumab 120 mg** | **Galcanezumab 240 mg** | **Placebo** |
| --- | --- | --- | --- |
| **Episodic Migraine** |  |  |  |
| **Overall 6 months, (N)** | 60.4% (436) | 58.9% (428) | 37.7% (875) |
| **Month 1**, **(n/N)** | 50.8% (221/435) | 47.1% (201/427) | 23.7% (207/872) |
| **Month 6**, **(n/N)** | 66.0% (246/373) | 63.9% (232/363) | 44.8% (324/724) |
| **Chronic Migraine** | **Galcanezumab 120 mg** | **Galcanezumab 240 mg** | **Placebo** |
| **Overall 3 months, (N)** | 30.7% (273) | 30.7% (274) | 17.8% (538) |
| **Month 1**, **(n/N)** | 26.4% (72/273) | 23.7% (64/270) | 11.0% (59/535) |
| **Month 3**, **(n/N)** | 35.2% (90/256) | 37.0% (97/262) | 24.7% (123/498) |
